# Effects of mulching on soil CO_2_ fluxes, hay yield and nutritional yield in a forage maize field in Northwest China

**DOI:** 10.1038/s41598-019-50475-8

**Published:** 2019-10-02

**Authors:** Ming Fan, Qiang Li, Enhe Zhang, Qinglin Liu, Qi Wang

**Affiliations:** 1 College of Agronomy, Gansu Agricultural University, Lanzhou, 730070 China; 2 College of Grassland Science, Gansu Agricultural University, Lanzhou, 730070 China

**Keywords:** Grassland ecology, Plant ecology

## Abstract

In arid areas of China, water shortage and heavy carbon emissions have been threatening agricultural sustainability and which has become a vital issue. A field experiment was conducted to explore how different mulching affects soil moisture and temperature, CO_2_ fluxes, forage-maize hay yield and nutritional value during 2 consecutive years: 2014 and 2015. The field experiment showed that mulching materials had distinct effects on soil moisture and temperature and CO_2_ fluxes. The soil temperature and CO_2_ fluxes were in order of common plastic film mulching (PFM) > bio-degradable mulch mulching (BMM) > no mulching (CK) > straw mulching (SM), while the soil moisture was in order of PFM > BMM > SM > CK over these two years. Compared with CK, hay yield respectively increased by 23.25%, 22.51% and 5.27% for PFM, BMM and SM, WUE increased by 35.60%, 32.34% and 10.88%, and the total nutrient yields increased by 17.75%, 21.35% and 6.95%, respectively. To sum up, in combination with ecology and environmental protection, bio-degradable mulch could replace common plastic film and bio-degradable mulch should be popular in future. As bio-degradable mulch is green non-pollution, it is conducive to the sustainable development of agricultural ecosystem.

## Introduction

Maize (*Zea mays L*.) plays an extremely essential role in China’s food security, while it is also an important raw materials for food, chemical, fuel, medicine and other industries^1^. In recent years, with the rapid development of regional economy and animal husbandry, the status of maize in the feed is increasingly rising, the long-standing concept that as a food crop only focuses on grain yield has also been broken, the biological yield and nutritional quality of maize are highly appreciated^2^.

Gansu hexi oasis irrigated area is the main grain producing area in western China, where obviously characterized by an arid climate, little precipitation, high potential evaporation and adequate solar and hot resources. There is mainly depending on surface water and groundwater irrigation^3^, water deficiency is the main factor affecting crop yield in this area. Mulching cultivation technology is to cover the soil surface with crop stubble, straw, gravel, sand, wood chips and plastic film. Studies have shown that mulching can affect the growth and the organic matter accumulation of plant by regulating soil moisture and temperature, while the soil CO_2_ fluxes was particularly responsive to mulching^4–10^. In order to alleviate the problem of water shortage, common plastic film mulching and straw mulching are widely used in agricultural production^11^. Common plastic film caused a large number of mulch residues which seriously pollutes the environment, hinders agricultural mechanization and threatens the health of feeding straw livestock such as cattle and sheep^12,13^. It also damages soil structure, impedes the transfer of soil moisture and nutrients, thus damages the agricultural environment and is not conducive to sustainable agricultural development^14^. Straw mulching has realized the reuse of agricultural waste, while the decomposition of the straw can produce allelochemicals to effect the growth of crop seedling^15^. In view of this, more and more researchers are paying attention to the development and utilization of bio-degradable mulch. Studies on bio-degradable mulching have focused on the effects on soil moisture, temperature and crop yield, but there are few studies on CO_2_ fluxes and nutritional value^16–18^. A field study was conducted to explore the effect of different mulching (common plastic film mulching, bio-degradable mulch mulching, straw mulching, no mulching) on soil CO2 fluxes, forage-maize hay yield and nutritional value in dry areas of China from 2014 to 2015.

## Materials and Methods

### Site description

**Figure 1:**
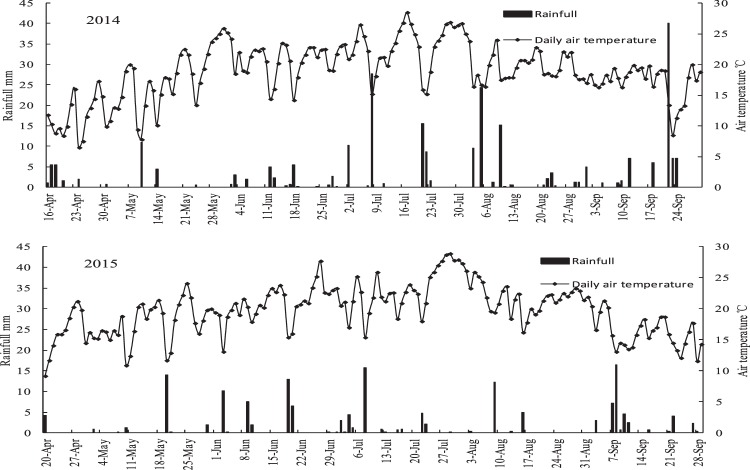
Rainfall and air temperature during growing seasons in 2014 and 2015.

The field experiment was carried out in 2014 and 2015 at the Wuwei experimental station of Gansu Agricultural University in an arid oasis region (37°96′N, 102°64′E). The station, located in the eastern part of the Hexi corridor of northwestern China, is in the temperate arid zone in the hinterland of the Eurasia Continent. Long term (30 years)average solar radiation is 6000 MJ m^−2^, annual sunshine duration is >2945 h, annual mean temperature is 7.2 °C with accumulated temperature above 0 °C >3513 °C and above 10 °C >2985 °C^9,10^. Mean annual precipitation is rarely greater than 156 mm, occurring mainly in the summer (Fig. 1), however, annual potential evaporation is greater than 2400 mm. The soil at the experimental site was classified as an Aridisol (FAO/UNESCO, 1988), and some of the properties are presented in Table 1. At the start of the experiment, the total nitrogen (N), Olsen P, and organic matter of the top (0–20 cm) soil was 0.78 g kg^−1^, 1.14 g kg^−1^ and 14.3 g kg^−1^, respectively.

**Table 1:** Some soil properties across 0–120 cm soil profile at Wuwei experimental station.

Depths Cm	Bulk density Mg m^−3^	Wilting point %	Field capacity %	Soil texture^a^	Particle size %^b^		
					sand	silt	clay
0–20	1.41	6.7	20.2	Silt loam	28.6	65.4	5.1
20–40	1.52	9.6	23.4	Silt loam	25.6	69.8	4.6
40–60	1.55	10.2	26.2	Silt	16.7	79.8	4.1
60–80	1.53	10.8	26.9	Silt	18.0	78.2	3.8
80–100	1.51	11.4	27.6	Silt loam	25.6	70.2	3.6
100–120	1.50	11.2	27.7	Silt loam	26.7	69.7	3.6

^a^Soil texture is determined by using the soil particle percentage.

^b^Soil particle fraction based on the USDA textural soil classification system.

### Experimental design and field management

The experiment was conducted with a randomized, complete block design. There were 4 treatments (common plastic film mulching (PFM), bio-degradable mulch mulching (BMM), straw mulching (SM) and no mulching (CK)) with 3 replicates constituting a total of 12 plots. Plot size was 8*5 m^2^. Forage-maize cultivar (Xianyu 335) was manually sown. The thickness of the common plastic film and the biodegradable mulching film was 0.008 mm. The common plastic film was manufactured by Shijiazhuang Yongsheng Plastic Plant Co Ltd, China, and bio-degradable mulch film was manufactured by BASF Co Ltd, Germany. The biodegradable mulch film was composed of starch and other bio-materials. Straw mulch used the wheat straw.

The forage-maize was seeded on April 16, 2014 and April 20, 2015, plant spacing was 30 cm and row spacing 40 cm, and the density was 82,500 plants ha^−1^. Before sowing, the plots were divided and the land was ploughed once and harrowed. According to local fertilizer application, a base fertilizer containing 150 kg P ha^−1^ and 40 kg K ha^−1^ was spread evenly over planting belts and then ploughed into top soil before sowing. In the whole growth period of maize, the nitrogen fertilizer was pure N 430 kg ha^−1^ (common urea, containing 46.4% of pure nitrogen). N fertilizer was used in 30%, 21%, 42% and 7% of the total fertilizer during feeding maize sowing, elongation stage, large-belling and blossom. The experiment used drip irrigation system to guarantee same time and amount for plots. The irrigation amount for seeding, elongation stage, large-belling, blossom and filling of forage-maize were 80, 110, 110, 110 and 110 mm, respectively, in 2014 and 2015. Forage-maize harvested on September 29, 2014 and October 1, 2015.

### Data collection

#### Soil temperature

Soil temperature in each plot was measured at an interval of 5 days from sow to harvest. Soil temperatures in the 5, 10, 15, 20, and 25 cm soil depths were measured using curved pipe geothermometer at 8, 14, and 18 o’clock on the measurement day.

#### Soil moisture content and evapotranspiration

Soil moisture content(%) in each plot was measured in difference growth periods of forage-maize. The Soil moisture content measuring depth was 120 cm and per 20 cm layer. Soil was taken out by soil drill, and filled into aluminum box. First, weighed the aluminum box and wet soil(W_1_) by electronic balance, then continued to the drying of 12 h in the constant temperature of 105 °C, until constant weight, weighed the dry soil and aluminum box(W_2_) by electronic balance, next, weighed the aluminum box(W3) by electronic balance, finally, calculated soil water content using the equation as follows:

$${\rm{\theta }} \% =({{\rm{W}}}_{1}-{{\rm{W}}}_{2})/({{\rm{W}}}_{2}-{{\rm{W}}}_{3})\ast 100$$

where θ is the soil water content; W_1_ is the weight of the aluminum box and wet soil, W_2_ is the weight of dry soil and aluminum box, and W_3_ is the weight of aluminum box.

Evapotranspiration was determined using the equation as follows^19^:

$${\rm{ET}}={{\rm{P}}}_{{\rm{C}}}+{\rm{I}}+{\rm{U}}-{\rm{R}}-{{\rm{D}}}_{{\rm{w}}}-\Delta {\rm{S}}$$

Where Pc is the effective precipitation (mm), determined by the USDA soil conservation services method; I is the irrigation quota (mm); U is the upward capillary flow from the root zone(mm); R is the runoff (mm); D_w_ is the downward drainage out the root zone (mm); and ΔS is the change of soil water stored in the 0–120 cm layer (mm). The upward and downward flows were measured previously at a nearby field, and these two items have been found to be negligible in this semiarid area^20,21^. Runoff was also negligible due to small rains, and irrigation was controlled by raised ridges between plots. Therefore, the reduced equation is as follows:

$${\rm{ET}}={{\rm{P}}}_{{\rm{C}}}+{\rm{I}}-\Delta {\rm{S}}$$

Water use efficiency (WUE, kg ha^−1^ mm^−1^) was calculated as the ratio of hay yield (kg ha^−1^) to ET.

#### Soil CO_2_ fluxes

Soil CO_2_ fluxes was measured using a CFX-2 system(Soil CO_2_ Flux System, CFX-2, PP System Hitchin, UK) connected with a proprietary respiration chamber. At 12 h before measurement, all crop residues and other litters on soil surface were removed, and a hole with diameter the same as the respiration chamber size was made on the maize strips. The chamber, with a sharp edging point at the bottom, was placed on the soil surface and then pushed to the depth of 20 mm. Measurements were made at three places randomly selected in each plot, 5 values were recorded for each place within 180 s, and the average value was used for each plot. The diurnal soil CO_2_ fluxes was measured at 2 h intervals from 8:00 am to 6:00 pm on the blossom, and the seasonal measurements were implemented in large-belling(2014-6-22, 2015-6-25), blossom(2014-7-26, 2015-7-24), filling (2014-8-12, 2015-8-13) and maturity (2014-9-26, 2015-9-29).

#### Forage-maize hay yield

Forage-maize in each plot was hand-harvested at maturity, and the forage-maize was air dried and weighed for hay yield in film patterns.

#### Forage-maize hay nutritional value

The crude protein was determined by semi-micro kjeldahl method^22^, crude fat was determined by residual method^22^, the crude fibers were determined by Cooking method of elimination^22^. While the nutritional yield was calculated as the product of nutrient content and hay yield.

### Statistical analysis

Data were analyzed using the Mixed model of Statistical Analysis Software (SPSS software, 16.0, SPSS Institute Inc., USA), with the treatment as the fixed effect and replicate as random effect. The mean separation procedure was Duncan’s multiple-range test. Due to significant treatment by year interactions for most of the variables evaluated in the study, the treatment effect was assessed for each year separately. All significances were declared at the probability level of 0.05, unless otherwise stated.

## Results

### Soil temperature

**Figure 2:**
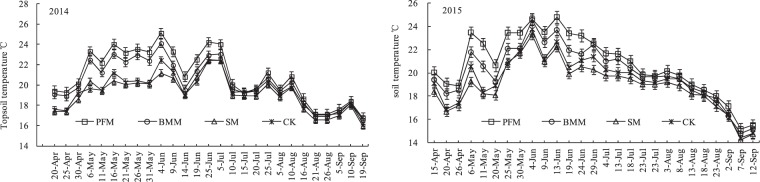
Soil temperature at 0–25 cm soil depth average in various treatments.

Topsoil temperatures increased with increasing air temperature at seedling stage, and decreased with rainfall and decreasing air temperature in early autumn. Topsoil (in 0–25 cm) average temperatures for various treatments during 2014 and 2015 maize growth period were shown in Fig. 2. In the early forage-maize growth (April to June), the topsoil temperature was significant between difference mulching treatments. While in the later maize growth (July to September), the topsoil temperature had no obvious difference between different mulching treatments. During the same maize growth period, the topsoil temperatures of PFM and BMM were significant higher than CK, while the topsoil temperature of SM was slight lower than CK. The average topsoil temperatures in forage-maize growing seasons were 20.9, 20.3, 19.2 and 19.4 °C in PFM, BMM, SM and CK, respectively in 2014, while were 20.8, 20.0, 19.0 and 19.3 °C in 2015. Compared with the CK, The average topsoil temperatures increased by 1.5 and 0.9 °C for PFM and BMM, respectively, and decreased by 0.2 °C for SM in 2014. But it increased by 1.5 and 0.7 °C for PFM and BMM, respectively, and decreased by 0.4 °C for SM in 2015.

### Soil water storage

**Figure 3:**
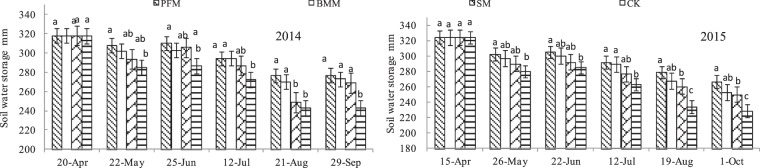
Soil water storage at 0–120 cm soil depth in various treatments.

Soil water storage is an important index to measure soil water balance. Normal variations in rainfall, evapotranspiration, root depth and mulching led to obvious differences in soil water storage (0–120 cm) during maize growing seasons for treatments in 2014 and 2015 (Fig. 3). From April to June in 2014 and 2015, the soil water storage was highest in forage-maize growth period. Except forage-maize sowing period, the soil water storage was in order of PFM > BMM > SM > CK. Compared with the CK, The average soil water storage increased by 8.20, 6.80 and 4.49% for PFM, BMM and SM in 2014, respectively, but increased by 9.59, 7.23 and 4.94% in 2015.

### Soil CO_2_ fluxes

**Figure 4:**
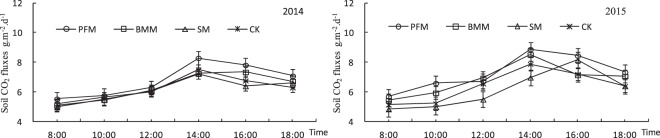
Diurnal variation soil CO_2_ fluxes in various treatments.

The diurnal variation soil CO_2_ fluxes for various treatments during 2014 and 2015 was presented unimodal curve, but the time of peak for unimodal curve was different in Fig. 4. The diurnal variation soil CO_2_ fluxes of unimodal curve peak for PFM, BMM and CK was afternoon 2 ‘clock, respectively, while SM was afternoon 4 ‘clock. Compared with the CK, The average diurnal variation soil CO_2_ fluxes increased by 8.94%, 1.44% and −1.92 for PFM, BMM and SM, respectively, While increased by 13.63%, 6.82% and −4.14% in 2015.

Soil CO_2_ fluxes for various treatments during 2014 and 2015 maize growing seasons was shown in Table 2. During different maize growth periods, soil CO_2_ fluxes was in order of blossom > large-belling > filling > maturity over two years. The average soil CO_2_ fluxes in maize growing seasons were 4.06, 4.01, 3.61 and 3.81 g m^−2^ d^−1^ in PFM, BMM, SM and CK, respectively in 2014, while were 4.75, 4.41, 3.95 and 4.09 g m^−2^ d^−1^ in 2015.

**Table 2:** Soil CO_2_ fluxes for forage-maize growing seasons in various treatments.

Years	Treatment	Soil fluxes g m^−2^ d^−1^			
		Large-belling	Blossom	Filling	Maturity
2014	PFM	5.80 ± 0.35a	6.74 ± 0.28a	3.74 ± 0.46a	1.71 ± 0.23a
	BMM	5.05 ± 0.21b	6.33 ± 0.20a	3.00 ± 0.28ab	1.69 ± 0.16a
	SM	4.89 ± 0.41b	6.12 ± 0.32a	2.04 ± 0.35b	1.38 ± 0.21a
	CK	5.01 ± 0.16b	6.24 ± 0.26a	2.35 ± 0.22b	1.64 ± 0.19a
2015	PFM	5.74 ± 0.23a	7.28 ± 0.37a	3.96 ± 0.25a	2.03 ± 0.21a
	BMM	5.46 ± 0.18a	6.84 ± 0.29a	3.59 ± 0.32a	1.76 ± 0.19a
	SM	5.19 ± 0.21a	6.14 ± 0.21b	2.90 ± 0.18a	1.57 ± 0.17a
	CK	5.16 ± 0.15a	6.40 ± 0.26ab	3.10 ± 0.21a	1.68 ± 0.22a
Average	2014	4.98 ± 0.28	6.36 ± 0.27	2.78 ± 0.33	1.61 ± 0.19
	2015	5.39 ± 0.19	6.67 ± 0.28	3.38 ± 0.24	1.76 ± 0.19

Different letters in same year of each column mean significantly difference at P < 0.05 according to Duncan’s multiple comparison test.

### Forage-maize hay yield and WUE

According to Table 3, the hay yield was in order of PFM > BMM > SM > CK in two years. The hay yield of PFM and BMM was significantly higher than SM and CK, but no significant differences were found between PFM and BMM, SM and CK over 2 years. The hay yield increased by 22.87%, 21.16% and4.09% for PFM, BMM and SM, respectively, in 2014, but increased by 23.63%, 23.85% and 6.45% in 2015 compared with the CK.

**Table 3:** Hay yield and WUE in various treatments.

Years	Treatment	Hay yield kg ha^−1^	ET mm	WUE kg ha^−1^ mm^−1^
2014	PFM	29700 ± 1608a	724 ± 18b	41.02 ± 2.06a
	BMM	29288 ± 2193a	737 ± 22ab	39.74 ± 1.83a
	SM	25163 ± 1584b	754 ± 15ab	33.37 ± 0.96b
	CK	24173 ± 2021b	792 ± 21a	30.52 ± 2.43b
2015	PFM	30700 ± 2354a	732 ± 15b	41.94 ± 2.83a
	BMM	30756 ± 1864a	746 ± 19b	41.23 ± 1.74a
	SM	26435 ± 2105b	767 ± 23ab	34.46 ± 2.29b
	CK	24832 ± 1360b	810 ± 26a	30.66 ± 2.31b
Average	2014	27081 ± 1851	752 ± 19	36.16 ± 1.82
	2015	27331 ± 1920	764 ± 21	37.07 ± 2.29

Different letters in same intercropping of each column mean significantly difference at P < 0.05 according to Duncan’s multiple comparison test.

Based on hay yield, the WUE was in order of PFM > BMM > SM > CK in two years. The WUE of PFM and BMM was significantly higher than SM and CK, but no significant differences were found between PFM and BMM, SM and CK over two years. The WUE increased by 34.40%, 30.20% and 9.34% for PFM, BMM and SM, respectively, in 2014, while increased by 36.80%,34.48% and 12.42% in 2015 compared with the CK.

### Forage-maize hay nutrient contents and nutritional yields

The crude protein and crude fiber content of SM was significantly higher than PFM, but no significant differences were found between others treatments in two years. While the crude fat content of treatments was no significant difference(Table 4). The crude protein content decreased by 5.26%, 2.42% and −3.09% for PFM, BMM and SM, respectively, in 2014, while decreased by 7.12%, 0.25% and −0.32% in 2015 compared with the CK. The crude fiber content decreased by 2.55%, 0.91% and −3.10% for PFM, BMM and SM, respectively, in 2014, while decreased by 5.78%, 0.71% and −0.18% in 2015 compared with the CK.

**Table 4:** Nutrient contents and nutritional yields in various treatments.

Years	Treat -ment	Nutrient contents %			Crude protein	Nutritional yields kg ha^−1^		
		Crude protein	Crude fat	Crude fiber		Crude fat	Crude fiber	Total yields
2014	PFM	6.08 ± 0.16b	2.22 ± 0.12a	25.53 ± 1.47b	1807 ± 83a	660 ± 23a	7583 ± 328a	10050a
	BMM	6.26 ± 0.09ab	2.28 ± 0.07a	25.96 ± 1.23ab	1835 ± 121a	667 ± 19a	7603 ± 234a	10104a
	SM	6.62 ± 0.13a	2.35 ± 0.09a	27.01 ± 0.98a	1665 ± 79b	592 ± 27b	6797 ± 298b	9055b
	CK	6.42 ± 0.11ab	2.32 ± 0.15a	26.20 ± 1.19ab	1552 ± 83b	561 ± 15b	6334 ± 312b	8447c
2015	PFM	5.83 ± 0.19b	2.23 ± 0.06a	26.53 ± 1.02b	1790 ± 102a	685 ± 12a	8145 ± 274a	10619a
	BMM	6.26 ± 0.23ab	2.28 ± 0.12a	27.96 ± 0.95ab	1927 ± 92a	700 ± 24a	8599 ± 364a	11226a
	SM	6.40 ± 0.08a	2.30 ± 0.17a	28.21 ± 0.84a	1665 ± 88b	608 ± 21b	7458 ± 295b	9731b
	CK	6.28 ± 0.19ab	2.29 ± 0.11a	28.16 ± 1.32ab	1559 ± 72b	569 ± 19b	6993 ± 383b	9121c
Average	2014	6.35 ± 0.12	2.29 ± 0.11	26.18 ± 1.21	1715 ± 91	620 ± 21	7079 ± 293	9414
	2015	6.17 ± 0.17	2.27 ± 0.12	27.72 ± 1.03	1735 ± 88	640 ± 19	7799 ± 329	10174

Different letters in same intercropping of each column mean significantly difference at P < 0.05 according to Duncan’s multiple comparison test.

Nutritional yields was the most direct index to judge forage value. The crude protein, crude fiber, and crude fat yields of BMM was highest in 2014 (1835, 667 and 7603 kg ha^−1^) and 2015 (1927, 700 and 8599 kg ha^−1^). The crude protein, crude fiber, and crude fat yields of PFM and BMM was significantly higher than SM and CK, but no significant differences were found between PFM and BMM, SM and CK in 2 years. In terms of total nutrient yields, PFM and BMM were significantly higher than SM, while SM was significantly higher than CK in 2014 and 2015. The total nutrient yields increased by 18.98%, 19.62% and7.19% for PFM, BMM and SM, respectively, in 2014, while increased by 16.43%, 23.08% and 6.69% in 2015 compared with the CK.

## Discussion

Water was the main factor that restricted agricultural and animal husbandry production in the west arid regions of China^23^. Mulching improved the plant growing environment by reducing soil moisture evaporation and regulating soil temperature. Li *et al*.^24^ observed that the average temperature in 5–25 cm tilth soil under common plastic film and biodegradable film mulching were 2.51–3.77 °C and 1.30–2.19 °C, respectively, which is higher than that of the uncovered ground. We found that the use of various mulching had distinct effects on soil temperature and soil water storage. With crops growing, full plant canopy was established during the middle and later growth stages which led to small differences of soil temperature among treatments. The average topsoil temperatures increased by 1.5 and 0.8 °C for PFM and BMM, respectively, and decreased by 0.3 °C for SM over two years. Meanwhile, maize was growth slowly and root distributed shallow in early time, with the rapid vegetative growth and reproductive growth of maize, soil water storage of treatments gradually decreased. Compared with the CK, the average soil water storage increased by 8.89, 7.01 and 4.49% for PFM, BMM and SM over two years, respectively. A field experiment using oats as an indicator crop showed that mulching materials had distinct effects on topsoil temperature^25^. Soil was the largest carbon sink in terrestrial ecosystems, while soil CO_2_ fluxes was impacted by soil water and heat. We found that mulching film increased soil fluxes, straw mulching decreased soil fluxes. The reason that mulching film increased topsoil temperature but straw mulching decreased topsoil temperature. The soil CO_2_ fluxes showed a seasonal variation and fluctuated with the soil and the atmospheric temperature for upland measured in Zigui, Three Gorges region^26^.

Several investigators have reported that mulching can improve crop yields and water fertilizer rate^27,28^. Zhang *et al*. observed that fields with recourse to common mulch and biodegradable film mulch showed an average increase in yield 19.23% and 17.82%, respectively, average WUE were 21.49% and 20.25% over two years^29^. We found that hay yield increased by 23.25%, 22.51% and 5.27% for PFM, BMM and SM, respectively, over two years compared with the CK. While WUE increased by 35.60%, 32.34% and 10.88%, respectively, over two years. Investigators have reported that film mulching was significantly higher than straw mulching for yield which was probably because film mulching reduced soil evaporation on ridges and augmented infiltration of rainwater and irrigation into soil^30^. The results of research showed that nutrient contents was in order of SM > CK > BMM > PFM, which was similar to the results of Pan *et al*. and Li *et al*. studied on maize and tobacco leaves^31,32^. Possibly because straw mulching could decompose and fertilize soil, which was beneficial for maize growth and organic matter transformation. The crude protein, crude fiber, and crude fat yields of BMM was highest in two years. Compared with CK, the total nutritional yields increased by 17.75%, 21.35% and 6.95% for PFM, BMM and SM, respectively. In agricultural and animal husbandry, we should consider not only the nutrient content of herbage but also calculate the nutrient yield.

Mulching was an important measure to increase agricultural yield in this area. However, common plastic mulch was not easy to degrade and resulting in environmental pollution. Therefore, biodegradable plastic mulch should be promoted in future agricultural production. As biodegradable plastic mulch was green and environmental friendly, it was conducive to the sustainable development of regional agriculture.

## Conclusions

The results of research showed that soil temperature and soil CO_2_ fluxes were in order of PFM > BMM > CK > SM, while the soil water storage were in order of PFM > BMM > SM > CK over two years. Hay yield increased by 23.25%, 22.51% and 5.27% for PFM, BMM and SM, respectively, compared with the CK over two years, while WUE increased by 35.60%, 32.34% and 10.88%, respectively. The total nutrient yields increased by 17.75%, 21.35% and 6.95%, respectively. To sum up, in combination with ecology and environmental protection, bio-degradable mulch could replace common plastic film and bio-degradable mulch should be popular in the future. As bio-degradable mulch was green and free-pollution, it was conducive to the sustainable development of agricultural ecosystem. However, this paper only focused on the changes of soil water, temperature, soil respiration, maize hay yield and nutrient yield after biodegradable plastic mulching. Next, we will study the degradation rate of biodegradable plastic film and the changes of soil physico-chemical properties after mulching.
